# Identification and functional characterization of novel xylose transporters from the cell factories *Aspergillus niger* and *Trichoderma reesei*

**DOI:** 10.1186/s13068-016-0564-4

**Published:** 2016-07-20

**Authors:** Jasper Sloothaak, Juan Antonio Tamayo-Ramos, Dorett I. Odoni, Thanaporn Laothanachareon, Christian Derntl, Astrid R. Mach-Aigner, Vitor A. P. Martins dos Santos, Peter J. Schaap

**Affiliations:** 1 Laboratory of Systems and Synthetic Biology, Wageningen University and Research, Stippeneng 4, 6708WE Wageningen, The Netherlands; 2 Enzyme Technology Laboratory and Integrative Biorefinery Laboratory, National Center for Genetic Engineering and Biotechnology (BIOTEC), National Science and Technology Development Agency, Thailand Science Park, 113 Pahonyothin Road, Pathumthani, 12120 Thailand; 3 Research Area Biochemical Technology, Institute of Chemical Engineering, TU Wien, Gumpendorfer Str. 1a, Vienna, Austria

**Keywords:** *Aspergillus niger*, *Trichoderma reesei*, Hidden Markov model, Xylose, Sugar porter, Transport kinetics, XltA, XltB, XltC, Str1, Str2, Str3

## Abstract

**Background:**

Global climate change and fossil fuels limitations have boosted the demand for robust and efficient microbial factories for the manufacturing of bio-based products from renewable feedstocks. In this regard, efforts have been done to enhance the enzyme-secreting ability of lignocellulose-degrading fungi, aiming to improve protein yields while taking advantage of their ability to use lignocellulosic feedstocks. Access to sugars in complex polysaccharides depends not only on their release by specific hydrolytic enzymes, but also on the presence of transporters capable of effectively transporting the constituent sugars into the cell. This study aims to identify and characterize xylose transporters from *Aspergillus niger* and *Trichoderma reesei*, two fungi that have been industrially exploited for decades for the production of lignocellulose-degrading hydrolytic enzymes.

**Results:**

A hidden Markov model for the identification of xylose transporters was developed and used to analyze the *A. niger* and *T. reesei* in silico proteomes, yielding a list of candidate xylose transporters. From this list, three *A. niger* (XltA, XltB and XltC) and three *T. reesei* (Str1, Str2 and Str3) transporters were selected, functionally validated and biochemically characterized through their expression in a *Saccharomyces cerevisiae* hexose transport null mutant, engineered to be able to metabolize xylose but unable to transport this sugar. All six transporters were able to support growth of the engineered yeast on xylose but varied in affinities and efficiencies in the uptake of the pentose. Amino acid sequence analysis of the selected transporters showed the presence of specific residues and motifs recently associated to xylose transporters. Transcriptional analysis of *A. niger* and *T. reesei* showed that XltA and Str1 were specifically induced by xylose and dependent on the XlnR/Xyr1 regulators, signifying a biological role for these transporters in xylose utilization.

**Conclusions:**

This study revealed the existence of a variety of xylose transporters in the cell factories *A. niger* and *T. reesei*. The particular substrate specificity and biochemical properties displayed by *A. niger* XltA and XltB suggested a possible biological role for these transporters in xylose uptake. New insights were also gained into the molecular mechanisms regulating the pentose utilization, at inducer uptake level, in these fungi. Analysis of the *A. niger* and *T. reesei* predicted transportome with the newly developed hidden Markov model showed to be an efficient approach for the identification of new xylose transporting proteins.

**Electronic supplementary material:**

The online version of this article (doi:10.1186/s13068-016-0564-4) contains supplementary material, which is available to authorized users.

## Background

Industrial production of chemicals and enzymes synthesized by fungi comprises a huge international market [1–3] and filamentous fungi such as *Aspergillus niger* and *Trichoderma reesei* have become two of the main workhorses of today’s industrial biotechnology. Their high enzyme secretory capacity has already been industrially exploited during the last decades and both species have the capacity to efficiently degrade and utilize second-generation lignocellulosic feedstocks [4]. Global climate change, fossil fuels limitations and breakthroughs in the advanced biofuels market have boosted the demand for robust and efficient microbial cell factories. In this regard, to enhance the enzyme-secreting ability of these fungi, efforts at multiple levels have been done: from studies at system level to understand the secretory process, to the use of more applied strategies focused on the improvement of protein yields [5, 6]. Nevertheless, access to sugars released from the complex polysaccharides is not only dependent on the ability of these fungi to secrete high titers of a complex mix of hydrolytic enzymes, but also on the presence of a large array of sugar porters, transport proteins that are capable of effectively transporting the constituent sugars into the cell.

Most of the current knowledge on sugar transporters in fungi comes from studies in the model organism *Saccharomyces cerevisiae*. *S. cerevisiae* is able to consume a limited set of mono- and disaccharides [7]. When the in silico proteome is explored with Pfam profile hidden Markov models (HMM) [8] for different transporter proteins, 73 proteins may be classified as major facilitator superfamily (MFS, Pfam ID: PF07690) transporters, of which 43 belong to the sugar porter (SP, Pfam ID: PF00083) subfamily. The superior capacity of lignocellulose-degrading fungi for sugar uptake seems clear from the number of sugar porters that are available. In the *A. niger* in silico proteome 469 proteins may be classified as MFS transporters of which 256 are included in the SP subfamily [9]. Using the same approach, we estimate that for *T. reesei* 235 proteins can be classified as MFS transporters of which 113 can be classified as SP.

To completely eliminate the ability of *S. cerevisiae* to use glucose as a carbon source 20 SP genes needed to be knocked-out [10]. This apparent redundancy enables the organisms to efficiently take up the available carbon source in response to a wide variety of conditions. In yeast not only sugar transporters contribute to this flexibility, but also a number of sensors that share with these transporters the same domain architecture, but have an additional domain to connect to intracellular signal transduction pathways [11, 12]. While glucose is always the preferred substrate, many of these yeast transporters have the ability to transport several other sugars including natively non-fermentable substrates like xylose. Members of the sugar porter subfamily are structurally similar in design, with 12 transmembrane segments organized in two distinct domains [13, 14] and only a limited number of amino acid side chains are believed to control binding and affinity [15, 16]. In search for more efficient xylose transporters, recent studies have focused on changing the functionality of known yeast transporters and it has been shown that it is possible to change the uptake rate, affinity and inhibition through a limited set of mutations [17–21]. Native industrially relevant xylose transporters that show high affinity and capacity for xylose uptake have been identified from *Escherichia coli*, *Pichia stipitis* and *Candida intermedia* [22–24]. Additionally several transporters from filamentous fungi have been reported to transport xylose, such as An25 and An29-*2* from *Neurospora crassa*, XtrD from *A. nidulans*, MstA from *A. niger* and Xlt1 and Str1 from *T. reesei* [25–28]. Of this list, only An25 and MstA have been biochemically characterized.

*A. niger* and *T. reesei* are natural xylose metabolizers. The aim of this study was to study their *modus operandi* in xylose transport. This was done through the identification, characterization and regulation analysis of a novel set of native transporters able to use xylose as a substrate. To select promising new candidates, a hidden Markov model specific for xylose transporters (HMM_xylT_) was developed, similarly to the HMM developed for glucose transporters [9]. With HMM_xylT_, the in silico proteomes of *A. niger* and *T. reesei* were mined for candidate xylose transporters and from each species three candidate xylose transporters were selected for experimental validation and biochemical characterization. All six transporters were able to transport xylose and between them they showed remarkable differences in terms of transcriptional expression regulation and substrate affinity and selectivity.

## Results and discussion

### *A. niger* and *T. reesei* in silico proteome mining

**Fig. 1:**
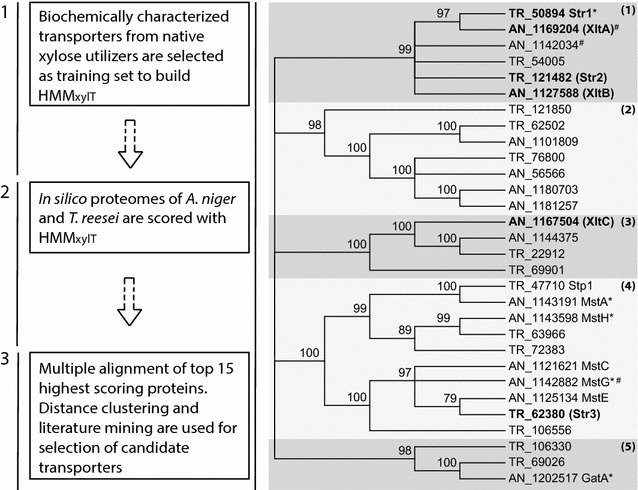
Approach for selection of candidate transporters  (steps 1, 2 and 3), and neighbor-joining distance tree of top 15 highest HMM_xylT_ scoring proteins from *A. niger* and *T. reesei*. Functionally validated xylose transporters were retrieved to train a HMM_xylT_, and used to analyze the in silico proteomes of *A. niger* and *T. reesei*. An analysis of the phylogenetic relationships of the top-scoring candidate transporters, taking into account the available literature, allowed the selection of candidate transporters that were subsequently characterized. Bootstrap values are indicated, nodes with bootstrap values <75 % are collapsed. Numbers indicate protein ID in JGI online genomes of *A. niger* ATCC1015 and *T. reesei* v2.0 [50, 66]. Proteins selected for further biochemical characterization are indicated in *bold* with protein names in brackets. AN_, *A. niger*; TR_, *T. reesei*; asterisks, previously experimentally validated transporters; hash symbol, the presence of a XlnR-binding motif as suggested by van Peij et al. [67] in the 1-kb upstream region of the encoding gene

Proteins belonging to the SP family are structurally similar, but share a low level of sequence identity [9] and only a limited number of amino acid side chains are believed to be involved in binding and affinity [15, 16]. As discussed in our previous work, HMMs can be used to more effectively segregate SP proteins based on their substrate than standard BLAST-based methods [9]. However, precision will largely depend on the availability of a consistent training set of previously characterized proteins with the function of interest. Given the data available in literature, we aimed to build a profile HMM as specific for xylose transporters as possible. To this end, we retrieved protein sequences from 24 functionally validated transporters that are able to transport xylose, with the additional requirement that they must originate from species that are naturally able to metabolize xylose (Additional file 1). Note that, when biochemically characterized, most of these xylose transporters either performed (at least) equally well on glucose, or were inhibited by the hexose, and thus the list is far from ideal. Nonetheless, we expected that residues fundamental for xylose transport are conserved in these proteins, ultimately making them xylose transporters as well. The HMM_xylT_ built from these sequences was used to mine the in silico proteomes of *A. niger* and *T. reesei* for putative xylose transporters. The complete workflow applied for the selection of candidate xylose transporters is presented in Fig. 1, Additional files 2 and 3 provide details on HMM_xylT_. The output list of top-scoring *A. niger* and *T. reesei* proteins was analyzed (Additional file 4), using the available literature, to search for the most promising candidates for xylose utilization. In this regard, the *A. niger* HMM_xylT_ output was compared with different transcriptome analysis studies where *A. niger* was grown in the presence of xylose, xylose–arabinose mixes, sugarcane bagasse, straw or willow [29–33]. The combined results of the HMM_xylT_ output and transcriptome analyses indicated that many of the new *A. niger* candidate transporters with a high HMM_xylT_ score were transcriptionally upregulated in, at least, one of these culture conditions (Additional file 5). Moreover, many of them were also possibly regulated by the XlnR and AraR transcription factors, which control hemicellulose utilization in *A. niger* [32]. This suggested that the top-scoring sugar porters within the *A. niger* HMM_xylT_ list contained a number of good candidates for further analysis. In the case of the *T. reesei* top-scoring sugar porters, only a few transcriptomic studies highlighting the expression modulation of hypothetical sugar transporters in hemicellulose degradation conditions are available. Still, in two recently published studies, analyzing the *T. reesei* transcriptomic response to wheat straw, many of the HMM_xylT_ top-scoring transporters were upregulated on this carbon source versus glucose [34, 35]. We also found that the function of three of the top-scoring transporters had been studied previously [25, 28, 36, 37]. One of them, TrStr1 (protID 50894) [28] was confirmed to function as xylose transporter, while Stp1 (protID 47710) and TrHxt1 (protID 22912) were associated to cellobiose and glucose utilization, respectively [36, 37].

### Phylogenetic clustering of top-scoring *A. niger* and *T. reesei* transporters

With the help of HMM_xylT_, a number of candidate xylose transporters from *A. niger* and *T. reesei* could be identified. Further analysis of the HMM_xylT_ output showed that the possible function of many high-scoring transporters could be linked to hemicellulose-associated sugars, particularly in *A. niger*. For this reason, three top 10 *A. niger* transporters AnXltA (protID 1169204), AnXltB (protID 1127588) and AnXltC (protID 1167504) were selected for further studies. For the selection of *T. reesei* xylose transporter candidates the top 15 highest scoring proteins from *A. niger* and *T. reesei* were aligned and a neighbor-joining distance tree was built from the pairwise distances (Fig. 1). Broadly, five protein clusters were observed. AnXltA and AnXltB are in cluster 1 and within this cluster *A. niger* XltA showed a strong clustering with *T. reesei* Str1 (protID 50894). Str1 was recently confirmed to be essential for pentose utilization and relevant in the induction of the hemicellulose utilization system [28], and since this transporter was not yet biochemically characterized it was selected for further study. In the same cluster, *T. reesei* protein 121482 (TrStr2) appeared to be a close homolog of *A. niger* XltB and thus TrStr2 was also selected for further analysis.

Several transporters from cluster 4 were previously studied, showing the relevance of this subgroup for sugar uptake [9, 37, 38]. Taking this into account, and the fact that MstA has been shown to have high affinity toward xylose, we decided to select another *T. reesei* representative of this cluster (TrStr3, ProtID 62380) for further studies.

### Engineering of a laboratory-evolved yeast strain for functional validation of xylose transporters

**Fig. 2:**
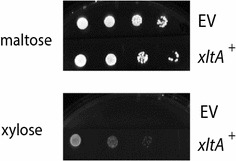
Functional validation of *A. niger* XltA as a xylose transporter, and of yeast strain EBY.XP as a xylose-utilizing strain. Serial dilutions of the EBY.XP *xltA* ^+^ strain, expressing the *A. niger* sugar porter gene cloned in expression vector p426HXT7-6His, were grown in minimal medium agar plates containing maltose or xylose 1 % (*w*/*v*). Agar plates were incubated at 30 °C for 5 days. EV: EBY.XP, carrying the empty expression vector p426HXT7-6His, was used as a negative control

*Saccharomyces cerevisiae* EBY.VW4000 is a strain unable to transport glucose and is frequently used for functional validation of sugar transporters [10]. For the purpose of ex vivo functional validation of the selected fungal transporters, strain EBY.VW4000 was genetically modified to be able to metabolize xylose as a carbon source, and laboratory-evolved for an enhanced growth on xylose. For this, strain EBY.VW4000, unable to grow on glucose, mannose, galactose or fructose as carbon source, was transformed with the plasmid pRH315, expressing the *P. stipitis*d-xylose reductase (XYL1) and xylitol dehydrogenase (XYL2) genes, and the *S. cerevisiae* xylulokinase (XKS1) gene [39]. An isolated transformant of the EBY.VW.4000 strain expressing the xylose utilization pathway (EBY.XP) was subsequently transformed with a plasmid expressing the *A. niger* hypothetical xylose transporter XltA (p426HXT7-6His-xltA). EBY.XP *xltA* transformants were isolated and the ability of XltA to confer them the capacity to grow in the presence of xylose was studied. Tenfold serial dilutions of exponentially growing cells from an *xltA* transformant were spotted on different minimal medium plates supplemented with either 1 % (*w*/*v*) maltose or 1 % (*w*/*v*) xylose. After an incubation period of 5 days, the *xltA* transformant strain showed ability to grow on xylose (Fig. 2). While this result confirmed that *A. niger* XltA is able to transport xylose, the ability of the transformant strain to grow on the pentose was poor. Poor growth of xylose-utilizing yeast transformant strains has been described before [25, 27, 28], and this could be due to metabolic imbalances as a result of different cofactor specificities of the heterologous genes XYL1 and XYL2 present in the modified *S. cerevisiae* strain [39]. To obtain a better growing xylose-utilizing host for routine identification of xylose transporters, the ability of the EBY.XP strain to metabolize xylose was improved through laboratory-evolution. For this, the strain was grown in agar plates containing selective minimal medium with 2 % (*w*/*v*) xylose, during long-term incubations (at least 2 weeks), at 30 °C. Once growing colonies were observed, they were pooled and re-plated again in 2 % (*w*/*v*) xylose plates. After two re-plating rounds, several single colonies showing relatively fast growth were selected and re-plated individually. Finally, a thus evolved strain showing the fastest growth, called Ag11, was selected for further research. Prior to further use as an optimized host for identification of new xylose transporters through functional complementation of transport, the expression vector carrying the *A. niger xltA* gene was cured from the Ag11 strain (see “Methods” section for details). The cured strain Ag11C3 was confirmed by its inability to grow in the absence of uridine, and in the presence of xylose as a single carbon source.

**Fig. 3:**
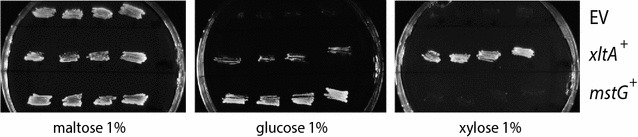
Functional validation of Ag11C3, a laboratory-evolved growth optimized derivative of the yeast strain EBY.XP. Growth of strain Ag11C3 expressing the *A. niger* *xltA* or *mstG* genes; or harboring the empty expression vector p426HXT7-6His (EV), in minimal medium agar plates containing maltose, glucose, or xylose 1 % (*w*/*v*). Four transformants per genetic background were tested. Agar plates were incubated at 30 °C for 7 days

To confirm that laboratory-evolved strain Ag11C3 could be successfully used as a host for the identification of new xylose transporters, the strain was retransformed with the p426HXT7-6His-xltA plasmid. Two Ag11C3-derived control strains were also constructed, one carrying the empty vector p426HXT7-6His, and one carrying the plasmid p426HXT7-6His-mstG, expressing a recently characterized glucose transporter from *A. niger* [9]. Four transformants per strain were isolated and, as can be observed in Fig. 3, only transformants carrying the p426HXT7-6His-xltA plasmid were able to grow on xylose. This result confirmed that the laboratory-evolved strain Ag11C3 can be used as host for the identification of transporters able to transport xylose. It also showed that the recently identified *A. niger* MstG glucose transporter is unable to support growth on xylose in this strain.

### Functional validation of *A. niger* AnXltA-C and *T. reesei* TrStr1-3 in yeast

**Fig. 4:**
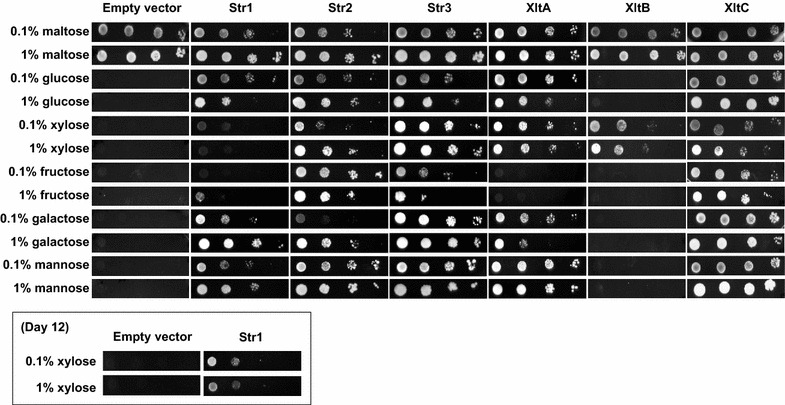
Heterologous expression and substrate utilization analysis of the *A. niger* sugar transporters XltA-C and *T. reesei* sugar transporters Str1-3. Ag11C3 transformants expressing *str1*, *str2*, *str3*, *xltA*, *xltB* or *xltC* were grown for 7 days at 30 °C in minimal medium agar plates containing a final concentration of 1 % (*w*/*v*) or 0.1 % (*w*/*v*) of the following sugars: maltose, glucose, xylose, fructose, galactose and mannose. EV: Ag11C3, carrying the empty expression vector p426HXT7-6His. *Insert* *str1* transformant grown for 12 days on xylose plates

Strain Ag11C3 was used to study the function of the selected *A. niger* and *T. reesei* sugar porters. Plasmids expressing *xltB*, *xltC*, *str1*, *str2* or *str3* were constructed using the p426HXT7-6His plasmid backbone and used to transform Ag11C3. Single-colony transformants were isolated from minimal medium agar plates containing 2 % maltose and the ability of XltA, XltB, XltC, Str1, Str2 and Str3 to support growth of the Ag11C3 transformant strains in the presence of xylose and in the presence of a number of other monosaccharides was studied using a plate assay. Tenfold serial dilutions of exponentially growing transformant cells were spotted on minimal medium plates supplemented with 1 % (*w*/*v*) or 0.1 % (*w*/*v*) of one of the following carbon sources: xylose, glucose, fructose, galactose, mannose and maltose (Fig. 4). All six previously selected transporters were functional as xylose transporters, as they all provided the Ag11C3 strain the ability to grow in xylose. Remarkable differences in the growth levels of the various transformants were observed, suggesting that the affinity of the transporters toward the pentose could be different. XltA, XltC and Str3 showed a good growth level in both high (1 %; *w*/*v*) and low (0.1 %; *w*/*v*) concentrations of xylose after 7 days, XltB and Str2 transformants showed a lower growth level in both xylose concentrations after the same incubation period, while growth of the Str1 transformant could only be clearly observed after a prolonged incubation time of 12 days. In addition, most of the transporters showed the ability to transport other monosaccharides. XltC, Str2 and Str3 were able to support growth of the Ag11C3 strain in the presence of all sugars tested, showing them to be the transporters with the broadest substrate specificity. XltA and Str1 showed a poor performance in the use of fructose as substrate but restored growth of Ag11C3 in the presence of glucose, galactose and mannose. The most remarkable substrate utilization profile was that of the Ag11C3-XltB transformant, which was only able to grow in the presence of xylose.

For a better insight in individual xylose affinities, the growth rates of transformants expressing of XltA, XltB, XltC, Str1, Str2 and Str3 were studied in liquid cultures in the presence of xylose (0.5 %; *w*/*v*) as sole carbon source, and in the presence of a mixture of xylose (0.5 %; *w*/*v*) and glucose (0.5 %; *w*/*v*) (Table 1). Additional file 6 shows growth curves of the transformant strains grown in minimal medium with the described sugar compositions. As was already observed in the plate assays, all transformant strains were able to grow in the presence of xylose as a sole carbon source. When comparing sugar uptake with growth curves, differences could be observed between the different transformants. With exception of the Str1 strain, in the presence of xylose as a sole carbon source growth started immediately after inoculation. In concordance to what has been observed in the plate assay, growth of Str1 transformant strain was observed only after 10 days. Some xylose consumption was observed by HPLC analysis, but due to the long lag time and poor growth this could not be properly quantified. Together, these results suggest that the xylose transport capacity of Str1 transporter is very low. In contrast, the Str3 transformant showed the fastest growth rate in the presence of the pentose, followed by XltC, Str2, XltB and XltA. At the end of the culturing period Str3 had consumed all xylose present in the media. Str2, XltC and XltB had used 50–57 % of the available xylose, while XltA used the 34 % (Table 1).

**Table 1:** Specific growth rate and sugar consumption by Ag11C3 transformants expressing XltA, XltB, XltC, Str1, Str2 and Str3

Ag11C3 strain	Xylose cultures		Xylose + glucose cultures		
	μ (h^−1^)	Total xylose consumed (%)	μ (h^−1^)	Total xylose consumed (%)	Total glucose consumed (%)
XltA	6.0 × 10^−3^ ± 1.7 × 10^−4^	33.8 ± 1.5	5.5 × 10^−3^ ± 1.1 × 10^−4^	ND	23.6 ± 0.4
XltB	6.3 × 10^−3^ ± 1.0 × 10^−4^	56.5 ± 1.8	6.2 × 10^−3^ ± 2.6 × 10^−4^	34.8 ± 0.6	15.7 ± 0.1
XltC	7.7 × 10^−3^ ± 3.6 × 10^−4^	50.0 ± 2.3	1.8 × 10^−2^ ± 7.6 × 10^−4^	ND	46.9 ± 5.8
Str1^a^	3.2 × 10^−3^ ± 3.6 × 10^−5^	NQ	4.8 × 10^−3^ ± 4.4 × 10^−5^	ND	ND
Str2	6.8 × 10^−3^ ± 1.8 × 10^−5^	50.6 ± 1.1	1.7 × 10^−2^ ± 3.8 × 10^−4^	ND	100.0
Str3	1.4 × 10^−2^ ± 1.5 × 10^−4^	100.0	1.8 × 10^−2^ ± 8.1 × 10^−5^	28.4 ± 0.3	100.0

μ = specific growth rate

*NQ* not quantifiable

*ND* not detectable

^a^Long lag phase on xylose

In the presence of the xylose and glucose mixture (0.5 + 0.5 %; *w*/*v*), each of the transformant strains behaved differently. In comparison to xylose cultures, growth of the Str1, Str2, Str3 and XltC transformant strains was enhanced by the addition of glucose, whereas growth of the XltB transformant was similar, and the XltA transformant grew less. This can be explained by different affinities of the respective sugar porters toward glucose and xylose. Str2 and Str3 transformant strains were able to use glucose efficiently (Table 1). In the presence of glucose, no xylose uptake could be determined with the Str2 transformant, whereas the Str3 transformant was able to use both carbon sources simultaneously. Xylose uptake by the XltC transformant was inhibited by glucose as well, but utilization of the hexose was not as efficient as observed for Str2 and Str3 (Table 1). In the presence of glucose Str1 initially grew faster, suggesting that Str1 could also transport glucose in the presence of the pentose, but growth levels when reaching the stationary phase were lower than observed in the cultures with xylose. Again sugar utilization could not be properly monitored by HPLC analysis suggesting a very low rate of glucose uptake as well.

When compared to the xylose medium, the growth rate of the Ag11C3 transformants expressing *A. niger* XltA and XltB was not higher in the mixed xylose–glucose medium, and in contrast to the *T. reesei* Str2 and Str3 transporters, glucose transport by *A. niger* XltA and XltB was not efficient. Moreover, the overall carbon uptake (glucose + xylose) in the sugar mix cultures was lower than that one observed for the xylose cultures (Table 1). This was also the case for the Ag11C3-XltC transformant. In the presence of glucose XltA was unable to transport xylose, while glucose was transported at a low rate (even lower than the xylose transport levels when the pentose was used as the sole carbon source). Inhibition of a xylose-specific transporter by glucose has been previously reported for the well-characterized XylE, from *E. coli* [13]. Even more remarkable was the performance of the XltB transformant in the mixed xylose–glucose medium: in the presence of glucose, xylose uptake was reduced but still two times higher than the uptake of glucose. Together with the plate assay study (Fig. 4), these results strongly suggest that XltB is a transporter specific for xylose.

To get more insights about both the biochemical properties and the biological role of these transporters, radiolabeled sugar uptake studies and transcriptional analysis of the respective coding genes were performed.

### Analysis of uptake kinetics by ^14^C-labeled sugar uptake studies

The growth data presented above effectively demonstrated that all the candidate transporters are able to transport xylose. Differences observed in growth rate and sugar uptake of yeast transformants expressing individual transporters suggested important differences in substrate specificity and uptake rate. We therefore measured the kinetics of xylose and glucose uptake of the six transporters. Transport assays were performed using a range of d-[1-^14^C]-xylose and d-[U-^14^C]-glucose concentrations. Initial uptake rates were calculated, fitted to the Michaelis–Menten model and used to estimate the appropriate kinetic parameters (*K*_m_ and *V*_max_) as previously described [40] (Table 2) (Additional file 7).

**Table 2:** Xylose and glucose initial uptake kinetics of fungal MFS transporters

Transporter	Xylose initial uptake kinetics		Glucose initial uptake kinetics		References
	*V*_max_ (nmol min^−1^ mg DW^−1^)	*K*_m_ (mM)	*V*_max_ (nmol min^−1^ mg DW^−1^)	*K*_m_ (mM)	
*AnXltA*	1.08 ± 0.05	0.09 ± 0.03	1.11 ± 0.15	0.07 ± 0.01	This study
*AnXltB*	0.10 ± 0.00	15.0 ± 4.50	ND	ND	This study
*AnXltC*	0.14 ± 0.01	4.71 ± 1.04	1.18 ± 0.15	0.11 ± 0.02	This study
*TrStr1*	0.04 ± 0.01	5.70 ± 0.19	0.14 ± 0.04	0.01 ± 0.00	This study
*TrStr2*	0.12 ± 0.02	6.18 ± 0.81	0.69 ± 0.19	0.05 ± 0.01	This study
*TrStr3*	0.46 ± 0.04	2.19 ± 0.29	1.31 ± 0.33	0.06 ± 0.01	This study
*AnMstA*	NC^a^	0.3 ± 0.1	NC	0.03 ± 0.01	[38]
GXS1	0.01 ± 0.00	0.08 ± 0.02	–	–	[17]
XUT3	0.01 ± 0.00	4.09 ± 1.08	–	–	[17]
Xyp29	0.61 ± 0.05	175.74 ± 21.36	ND	ND	[26]
*An25*	0.69 ± 0.04	55.96 ± 9.37	ND	ND	[26]
SUT1	132.0 ± 1.0	145.0 ± 1.0	45.0 ± 1.0	1.5 ± 0.1	[23]
SUT2^**+**^	41.0 ± 1.0	49.0 ± 1.0	3.3 ± 0.1 28.0 ± 4.0	1.1 ± 0.1 55 ± 11.0	[23]
SUT3^**+**^	87.0 ± 2.0	103.0 ± 3.0	3.7 ± 0.1 22.0 ± 0.1	0.8 ± 0.1 31.0 ± 0.1	[23]
Ag11C3	ND	ND	ND	ND	This study

Ag11C3; laboratory-evolved xylose-utilizing strain, transformed with the empty vector p426HXT7-6His

Italics: originating from filamentous fungi

**+** Two glucose transport components were reported

− Not determined

*ND* not detectable

*NC* not comparable

^a^Values were reported in an incompatible unit (nmol min^−1^ mg protein^−1^)

The affinity for xylose of the six transporters ranged from 90 μM (XltA) to 15 mM (XltB), being, in most cases, higher than those reported for most of the fungal xylose transporters characterized to date, with values between 80 μM and 150 mM [23, 24, 26, 38]. *A. niger* XltA showed to have a very high affinity for xylose (0.09 ± 0.03 mM), which is even higher than reported for the *E. coli* xylose transporter XylE (0.47 mM) [20, 22], and the *A. niger* high-affinity sugar transporter MstA (0.3 ± 0.1 mM) [38]. The transporters XltC (4.71 ± 1.04 mM), Str1 (5.70 ± 0.19 mM), Str2 (6.18 ± 0.81 mM) and Str3 (2.19 ± 0.29 mM) showed a high affinity toward xylose within the same order of magnitude, whereas XltB (15.00 ± 4.50 mM) had a slightly lower affinity. Regarding glucose transport characteristics, five out of six transporters showed a very high affinity for the hexose, ranging from 13 μM (Str1) to 108 μM (XltC). These are, in all cases, in the same range as reported for other *A. niger* high-affinity glucose transporters [9, 38]. It is a common feature of reported glucose/xylose transporters to show higher affinity for the hexose than for the pentose [26], with differences of around two orders of magnitude [20]. This was observed for the three *T. reesei* transporters characterized in this study, while the *A. niger* XltC affinity toward glucose was around 50 times higher than toward xylose (Table 2). However, *A. niger* XltA showed approximately the same high affinity for xylose (0.09 ± 0.03 mM) as for glucose (0.07 ± 0.01 mM). Also, XltA was able to transport both sugars at the same rate. In contrast, uptake of radiolabeled glucose by XltB was not detected, as was previously described for the *P. stipitis* Xyp29 and the *N. crassa* An25 xylose transporters [26]. This result was in concordance with the inability of XltB to support the growth of the Ag11C3 strain in the presence of glucose as a sole carbon source. The biochemical characteristics of the six studied transporters, plus those reported previously [9, 37, 38], revealed the wide range of glucose and xylose uptake systems featured by these fungi. The initial uptake kinetics of both sugars displayed by XltA and XltB suggested a possible biological role for these transporters in xylose utilization.

### Transcriptional analysis of *T. reesei str1*-*3* and *A. niger xltA*-*C*

**Fig. 5:**
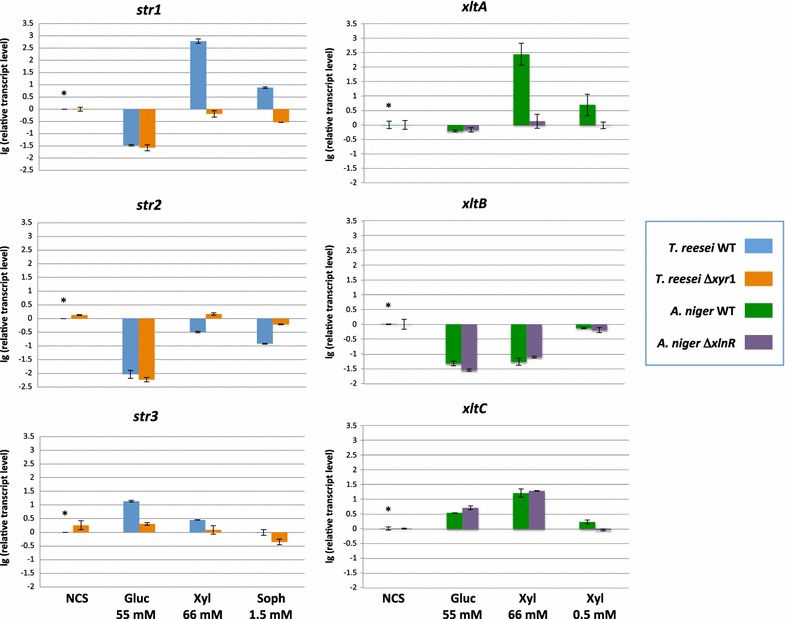
Transcriptional analysis of *T. reesei str1*-*3* and *A. niger xltA*-*C*. Samples were taken 3 h after mycelium transfer to the different culture conditions, and expression analyses were performed by RT-qPCR. Normalization of the expression data was done using the histone-like gene “*hist*” transcript (gene ID 207921) for *A. niger*, and genes *sar1* and *act1* for *T. reesei*. Results are given as relative transcript ratios in logarithmic scale (lg). The values provided in the figures are means of two biological replicates. Transcript levels always refer to the reference sample, indicated with an *asterisk*

To shed more light about their possible biological role on xylose uptake, a transcriptional analysis, performed via RT-qPCR of the respective transporter coding genes, was done. For this, the expression of *A. niger xltA*, *xltB* and *xltC*; and *T. reeseistr1*, *str2* and *str3* was studied, in different culture conditions, in both wild-type and *xlnR*/*xyr1* mutant strains which harbor an inactivated transcriptional activator of the xylanolytic system (Fig. 5). The samples for expression analysis of the *A. niger* genes were obtained from mycelium grown in cultures containing minimal medium plus 55 mM glucose, 66 mM xylose, 0.5 mM xylose, or no carbon source (NCS), whereas *T. reesei* samples were isolated from cultures on minimal medium containing one of the following carbon sources: 55 mM glucose, 66 mM xylose, 1.5 mM sophorose, or NCS.

In case of the *A. niger* transporter genes *xltA* was strongly induced by high xylose concentrations, and its expression levels were also significantly higher in lower xylose concentrations when compared to the rest of the conditions studied (Fig. 5). XlnR is a transcriptional activator of the xylanolytic system in *A. niger* [41]. In the *xlnR* mutant strain, *xltA* transcript levels dropped dramatically in the presence of low and high xylose concentrations, while they were kept at the same level in the NCS and glucose conditions. This indicates a clear role of XlnR in the transcriptional regulation of *xltA*, and thus, a role of XltA in xylose uptake by *A. niger.* The *xltB* gene expression was apparently not xylose dependent as it was repressed in the presence of high concentrations of both glucose and xylose, and its expression levels in the presence of low xylose concentrations were slightly lower than in the NCS condition. Also, the expression profile of *xltB* was similar in the wild-type and the ∆*xlnR* strain. Therefore, a role of XlnR in the regulation of this gene in the studied conditions could be discarded. According to *xltB* expression profile, XtlB seems to be relevant in *A. niger* when there is a low availability of carbon sources in the environment. In the uptake kinetics studies, the transport of low xylose concentrations by XltB could be determined, but it was not possible for low glucose concentrations. Thus, the substrate specificity, and the higher affinity for xylose than for glucose shown by XltB, together with its preferential expression at low carbon source concentrations, indicates that this transporter could have a role for xylose transport in the fungus independent of XlnR activation. The expression levels of *xltC* were, in contrast, higher when high sugar concentrations were present in the medium. They were particularly high in the xylose 66 mM condition. The role of XlnR on the transcriptional regulation of this gene was not clear, as its expression levels in the mutant strain were only slightly reduced in the xylose 0.5 mM condition, but not reduced at all in the xylose 66 mM condition.

Regarding the T*. reesei* genes, *str1* expression was strongly induced by xylose, in a similar way to *A. niger xltA*, and also by sophorose, which is also an inducer of the *T. reesei* xylanolytic system [42]. In addition, it was clearly transcriptionally regulated by Xyr1, encoding the main regulator of the xylose metabolism in *T. reesei* [43]. Despite Str1 low efficiency in xylose transport, the transcriptional behavior of its coding gene, and the recent findings reported by Huang et al. [28], suggests an important role for the transporter in xylose utilization. In the wild-type and ∆*xyr1* strains, *str2* was preferentially expressed in resting cell conditions and strongly down-regulated in the presence of 55 mM glucose. In case of the xylose and sophorose conditions, *str2* expression levels were slightly higher in the *xyr1* deletion strain. The *str3* gene did not seem to be specifically induced by xylose, being its expression levels higher in the presence of glucose (Fig. 5). In the absence of Xyr1 (∆*xyr1* strain), *str3* expression levels dropped in the presence of xylose and sophorose, but the same was observed in the glucose condition, while its expression was slightly increased when no carbon source was present. Although a role of Xyr1 in *str3* regulation cannot be discarded, the obtained results are not conclusive enough. The higher *str3* expression levels observed in the presence of 66 mM xylose and especially in 55 mM glucose, when compared to low concentrations of sophorose and the NCS condition (wild-type strain), do suggest a role for Str3 in the uptake of high sugar concentrations. This hypothesis is also in agreement with the glucose and xylose transporting behavior shown by the Str3-expressing yeast strain constructed in this study.

### Characteristics defining AnXltA-C and TrStr1-3 xylose transporters

**Fig. 6:**
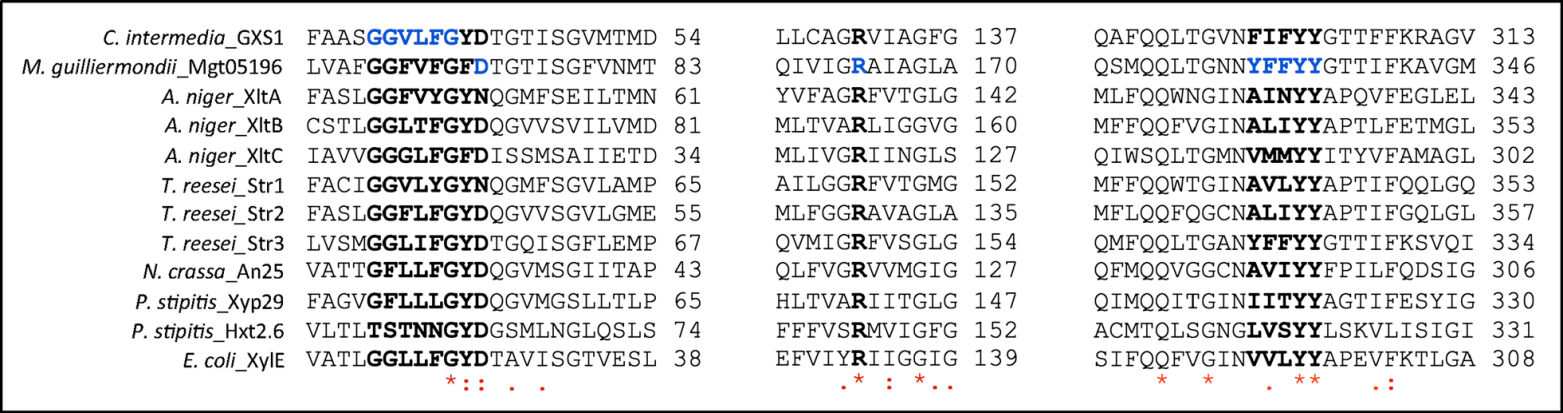
Conservation of motifs and residues reported to be relevant for xylose transport. A multiple sequence alignment of known xylose transporters, including *A. niger* XltA-C and *T. reesei* Str1-3 was constructed using the EMBL-EBI Clustal Omega tool [68]. Reported relevant motifs and residues are in *bold*; amino acids highlighted in blue indicate the transporter where motifs/residues were described to be relevant for xylose transport

Xylose was a substrate for all six different transporters, suggesting that the HMM_xylT_ may have captured residues discriminating for glucose–xylose porters. With the aim of pinpointing these discriminating residues, that should be conserved in the novel xylose transporter proteins, we used an MSA of all transporters used for HMM_gluT_ (constructed in a previous study, [9]) and HMM_xylT_ (this study), plus the newly identified *A. niger* and *T. reesei* transporters (Additional file 8). A number of motifs and residues from fungal sugar porters have been recently reported to be relevant for xylose transport by different studies, and the amino acid sequences of AnXltA-C and TrStr1-3 were analyzed for the presence of these motifs (Fig. 6). Wang and collaborators suggested the relevance of the aromatic residue enriched motif YFFYY (332–336), present in the transmembrane section 7, for the xylose transport capacity of Mgt05196p from *Meyerozyma guilliermondii* [44]. TrStr3, which showed the highest xylose transport capacity in this study, also contains the YFFYY (320–324) peptide. The same motif is present as well in a number of sugar porters (MSA), but it is not completely conserved in transporters that are exclusive for xylose as XylE from *E. coli*, An25 from *N. crassa* or Xyp29 (also called Xut6 and Stl12) from *P. stipitis*: the first three aromatic residues from the YFFYY motif are substituted by aliphatic ones in XylE and An25. This was also found to be the case in AnXtlB (ALIYY); AnXltC (VMMYY) (although not strictly an aliphatic residue, methionine is usually considered as such, since its sulfur group is not reactive); TrStr1 (AVLYY); and TrStr2 (ALIYY). In addition, the same motif in AnXltA (AINYY) had a polar residue (N) at the third position. Although most of the functionally validated sugar transporters contain a nonpolar (aromatic or aliphatic) amino acid at that position, several sugar porters, including the xylose transporters Hxt2.6 (LVSYY) and Xyp 29 (IITYY) from *P. stipitis*, contain a polar residue as well. The Mgt05196p residues aspartate D72 and arginine R164, also suggested to be crucial for xylose transport [44], were found to be conserved in AnXltB, AnXltC, TrStr2 and TrStr3. AnXltA and TrStr1 had the arginine conserved, but both contained an asparagine residue at the aspartate 72 of Mgt05196p. Since asparagine and aspartate are similar amino acids, both might have the same function at that particular position.

The motif GG/FXXXG, present at the first transmembrane span of sugar porters and highly enriched in those that confer growth on xylose [18], was also found to be present in AnXltA-C and TrStr1-3 (Fig. 6). Knoshaugh and collaborators [45] recently highlighted the variability of this motif, that allow to distinguish between xylose transporters (GGLXXGYD/N), arabinose transporters (XGXXFGFD) and glucose transporters (GGFXFGWG). This motif was subjected to protein engineering through saturation mutagenesis in the *C. intermedia* glucose–xylose symporter 1 (GXS1) [18]. In GXS1, the wild-type motif is GGVLFG (36–41). Saturation mutagenesis for each of the three variable residues (V38, L39, F40) produced changes in selectivity and efficiency of monosaccharide transport by GXS1. Residues V38 and F40 were found to be involved in carbon source selectivity, and L39 in controlling substrate transport efficiency. Interestingly, some of the most significant V38 substitutions were also found in the here studied transporters. V38F, that almost completely attenuated glucose exponential growth rate while amplifying exponential xylose growth rate by 50 %, was found in XltA and Str2. V38L, that increased the exponential xylose growth rate by 73 % without altering glucose exponential growth rate significantly, was found in XltB and Str3. V38G, which also produced a positive effect on xylose transport, was found in XltC. The L39 substitutions resulted in a general or differential attenuation of GXS1 transport function. L39I, found to be responsible for a specific attenuation of glucose transport in favor of xylose transport, was found in Str3. The L39V substitution, present in all three *P. stipitis* high-velocity xylose transporters SUT1-3 [23], was also found in XltA. Regarding the GXS1 F40 residue, XltB, XltC, Str2 and Str3 have the same amino acid at the same position, whereas XltA and Str1 contain a Y. The F40Y substitution in GXS1 produced an attenuating effect on the transport of xylose and other monosaccharides, indicating that the particular tyrosine residue could have the same role in XltA and Str1. This could explain, in part, the low efficiency displayed by Str1 on xylose transport, and provides a hint on how the transport capacity of both transporters could be improved.

Regarding the presence of amino acids that have been shown to be key at certain positions for glucose affinity in yeast sugar porters, like the threonine 219/213 and the asparagine 376/370 of *S cerevisiae* Gal2/Hxt7 [20, 46], interesting features were also found. Only XltC, Str2 and Str3, that were the most efficient glucose transporters in the presence of xylose (Table 1), keep both residues conserved; while XltB and Str1, that showed the lowest glucose transport capacity, have different residues in both positions.

In summary, the amino acid sequence analysis of the six transporters showed that all of them harbor motifs and residues previously associated to fungal xylose transporters. This fact, in combination with the experimental evidences provided, indicates a role of these transporters in xylose uptake.

## Conclusions

In this study, computational and experimental approaches were successfully combined for the identification and characterization of xylose transporting proteins from the industrial cell factories *A. niger* and *T. reesei*. Comparing the HMM_xylT_ output with recently published transcriptome studies, also taking into account phylogenetic distance relationships, was a good strategy to link a specific group of MFS porters with the utilization of lignocellulosic feedstocks. Using the mentioned methodology, five putative xylose transporters (XltA, XltB, XltC, Str2, Str3), and the recently identified xylose transporter Str1, were selected and successfully validated as xylose transporters. All of them displayed significant differences in their substrate specificity and biochemical properties, being XltA and XltB of particular interest, due to the high affinity for xylose of the former, and the narrow substrate specificity of the latter. Also, new insights about the regulation, at transcriptional level, of xylose utilization by *A. niger* and *T. reesei* were found, the most remarkable being that xylose uptake is not completely controlled by the XlnR/Xyr1 regulon.

To the best of our knowledge, this is the first study on the functional validation and characterization of sugar porters with their biological role specifically associated to xylose transport in *A. niger*. Also, the biochemical characterization of xylose transporters in *T. reesei* is reported for the first time. In summary, this study contributes to a better understanding of xylose utilization by two relevant industrial filamentous fungi, and provides new tools for strain engineering in fungi.

## Methods

### Construction of a xylose hidden Markov model

The protein sequences used to build HMM_xylT_ were obtained from the UniProt database [47], and aligned using the PRALINE structural alignment tool [48] with the same parameters as described for the hidden Markov model constructed in our previous work [9]. HMM_xylT_ was built using the HMMER v3.0 tool [49]. The *A. niger* ATCC1015 [50] and the *T. reesei* Rut-C30 [51] proteomes, which were used for the in silico analysis, were downloaded from the JGI database [52].

### Strains and growth conditions

*Escherichia coli* DH5α [*endA*1, *hsdR*17, *gyrA*96, *thi*-1, *relA*1, *supE*44, *recA*1, Δ *lacU*169 (Φ80 *lacZ*ΔM15)] was used for cloning experiments and plasmid propagation. It was grown at 37 °C on an LB medium (1 % tryptone, 0.5 % yeast extract, 1 % NaCl; *w*/*v*), with 100 μg mL^−1^ ampicillin when required for transformants selection.

*A.niger* N400 (CBS 120.49), NW199 (*fwn*A6, *leu*A5, *gox*C17, *pyr*A6; ∆*xlnR*::pIM240) [53] and *T. reesei* QM6a∆*tmus53* (ATCC 13631) [54] and QM6a∆*tmus53*∆*xyr1* (ATCC 13631) [55] strains were used in mycelium transfer experiments for the MFS genes transcriptional analysis. The *A. niger* and *T. reesei* strains were maintained on complete medium agar [56] and malt extract agar (MEX), respectively, at 30 °C.

Mycelium transfer experiments of both fungal species were performed in a similar way, using liquid cultures in Erlenmeyer flasks on a rotary shaker. The *A. niger* strains were pre-cultured at 30 °C and 200 rpm, during 18 h, in minimal medium containing 4.50 g L^−1^ NaNO_3_, 1.13 g L^−1^ KH_2_PO_4_, 0.38 g L^−1^ KCl, 0.38 g L^−1^ MgSO_4_·7 H_2_O, 750 μL L^−1^ of Vishniac solution, and 100 mM sorbitol [56, 57]. Equal amounts of water-rinsed mycelium were transferred to minimal medium with the following carbon source compositions: 55 mM d-glucose, 66 mM d-xylose, 0.5 mM d-xylose or no carbon source (NCS). The initial pH of the medium in all conditions was set at 6.0. The *T. reesei* strains were pre-cultured in Mandels–Andreotti (MA) medium [58], containing 1 % (*w*/*v*) glycerol as the sole carbon source, at 30 °C and 180 rpm for 22 h. Pre-grown mycelia were washed, then equal amounts were resuspended in MA media containing 55 mM d-glucose, 66 mM d-xylose, 1.5 mM sophorose or in medium without carbon source (NCS). In both experiments, 3 h after mycelium transfer samples were taken and quickly washed, dried with a single-use towel, snap-frozen with liquid nitrogen and stored at −80 °C until further processing. Two biological replicates per condition were studied in all cases.

The *S. cerevisiae* strain EBY.VW4000 (CEN.PK2-1C *hxt13Δ*::*loxP*; *hxt15::ΔloxP*; *hxt16Δ*::*loxP*; *hxt14Δ*::*loxP*; *hxt12Δ*::*loxP*; *hxt9Δ*::*loxP*; *hxt11Δ*::*loxP*; *hxt10Δ*::*loxP*; *hxt8Δ*::*lox*P; *hxt514Δ*::*loxP*; *hxt2Δ*::*loxP*; *hxt367Δ*::*loxP*; *gal2Δ*; *stl1Δ*::*loxP*; *agt1*::*loxP*; *ydl247wΔ*::*loxP*; *yjr160cΔ*::*loxP*) [10], previously transformed with the plasmid pRH315 [39], expressing the d-xylose reductase and xylitol dehydrogenase from *P. stipitis*, and the *S. cerevisiae* xylulokinase, was used as a xylose-utilizing strain for the characterization of sugar transporters. It was grown at 30 °C and maintained in the solid complete medium containing 10 g L^−1^ of yeast extract, 20 g L^−1^ of peptone and 20 g L^−1^ of maltose. The EBY.VW4000-derived strains obtained in the present study were grown in the liquid minimal medium containing 6.7 g L^−1^ of yeast nitrogen base with ammonium sulfate (Difco), 20 g L^−1^ of maltose, supplemented with leucine (30 mg L^−1^) and histidine (20 mg L^−1^). Growth rates (μ) of the Ag11C3 transformants, during time course cultivations, were calculated using the O.D. values obtained from *T* = 0 until the cultures reached stationary phase.

### Construction of *S. cerevisiae* Ag11C3 transformants expressing *A. niger* and *T. reesei* genes

The coding sequence of the genes *xltA*, *xltB*, *xltC*, *str1*, *str2* and *str3* was obtained through PCR amplification of *A. niger* and *T. reesei* cDNA samples, respectively. The coding sequence of the gene *xltA*, digested with *Spe*I and *Xho*I was cloned on the *S. cerevisiae* expression vector p426HXT7-6His [59], previously linearized with *Spe*I and *Xho*I, under the control of the constitutive promoter HXT7_p_ and the terminator CYC1_t_. The *xltB*, *xltC*, *str1*, *str2* and *str3* cDNA sequences were amplified with oligonucleotides containing 40 additional base pairs, corresponding to the p426HXT7-6His cloning site, and were cloned to the vector through yeast-mediated recombination [60]. Primer sequences and plasmids used in this study are provided in Additional file 9. The Ag11C3 yeast strain transformation was performed as previously described [61].

The curing of the p426HXT7-6His-xltA plasmid, containing the URA3 selection marker, from the Ag11 strain was done by growing the transformant in minimal medium plates containing maltose and uracil, thereby relieving the plasmid selective pressure. After three re-plating rounds in maltose-uracil plates, p426HXT7-6His-xltA cured strains were isolated through their growth on minimal medium plates containing maltose, uracil and 5-fluoroorotic acid (FOA). FOA is a commonly used agent to select for the absence of the URA3 gene in yeast strains [62]. A single colony growing on the FOA plate, named Ag11C3, was selected for its use as host for the expression of new xylose transporter candidates.

### Sugar analyses

Xylose and glucose present in the *S. cerevisiae* culture supernatants were quantified by high-pressure liquid chromatography (HPLC) analysis. The samples were centrifuged at maximum speed in a benchtop centrifuge for 10 min and analyzed on a Dionex ICS-5000+ instrument (Thermo Scientific), equipped with a CarboPac MA1 column. Separation was performed by isocratic elution with 480 mM NaOH, at a flow rate of 0.4 mL min^−1^ for 35 min.

### Analysis of uptake kinetics by ^14^C-labeled sugars’ uptake studies

Sugars’ uptake assays were performed as described [63], with minor adjustments. A pre-inoculum (50 mL) of Synthetic Enhanced medium (SE; 6.7 % (*w*/*v*) YNB w/o amino acids (Difco) + 20 mg L^−1^l-arginine and l-methionine), containing appropriate amino acids, and 2 % (*w*/*v*) maltose as a carbon source, was inoculated and incubated for 48 h (30 °C, 225 rpm). The pre-inoculum was then transferred to 200 mL of fresh SE-medium, and after 48-h incubation, transferred to 500 mL fresh SE-medium. After 24-h incubation, cells were harvested by centrifugation (4000*g*; 10 min) and washed with 50 mL ice-cold ultrapure water. Cells were then washed and resuspended in ice-cold 100 mM phosphate-buffered saline (PBS), pH 6.5, to an OD_600_ of approximately 500, divided in 40-μL aliquots, and kept on ice.

Aliquots were incubated for 5 min at 30 °C in a heat block with vigorous shaking before uptake assay was started. To start the reaction, 10 μL of a 5 times concentrated d-[1-^14^C]-xylose or d-[U-^14^C]-glucose solution (Campro Scientific) was added. After exactly 20 s, the reaction was stopped by the addition of 1 mL of appropriate ice-cold quenching buffer (100 mM PBS, pH 6.5, with 500 mM unlabeled d-xylose or d-glucose), followed by vacuum filtration (0.45 μm HV filters, 1225 sampling manifold, Millipore), and two subsequent washing steps with 5 mL of ice-cold quenching buffer. After 5 min of drying in the vacuum manifold, the filters were transferred to scintillation vials with 4 mL scintillation liquid (Ultima Gold, Perkin Elmer), and activity was counted (Packard Tricarb 1600TR). All reactions were performed in triplicates. All values were corrected using triplicate negative control measurements without incubation, where the quenching solution was added prior to the addition of labeled substrate. Uptake rates at two typical substrate concentration ranges were measured; 1–100 μM and 0.1–40 mM. Substrate solutions with an activity of approximately 5–5000 Bq μL^−1^ were used. To determine kinetic parameters *K*_m_ and *V*_max_, the data were fitted to the Michaelis–Menten model using the least squares method.

$$V = \frac{{V_{ \text{max} } \cdot [S]}}{{K_{\text{m}} + \left[ S \right]}}$$

### Transcriptional analysis of *A. niger* and *T. reesei* genes

RNA isolation from *A. niger* [9] and *T. reesei* [42] mycelium was done as described previously. Reverse transcription, quantitative PCRs and calculations were performed following the protocols and instruments described in Mach-Aigner et al. [64]. Primer sequences are provided in Additional file 9. Cycling conditions and control reactions were performed as described previously [65]. Normalization of the expression data was done using the previously described histone-like gene “*hist*” transcript (gene ID 207921) [64] for *A. niger*, and genes *sar1* and *act1* for *T. reesei* [65].

## Additional files

10.1186/s13068-016-0564-4 Protein sequences of 24 functionally validated xylose transporters used to train the profile HMM_xylT._

10.1186/s13068-016-0564-4 Structure based multiple sequence alignment of 24 verified xylose transporters_._

10.1186/s13068-016-0564-4 HMM_xylT_ used for the identification of *A. niger* and *T. reesei* xylose transporters.
10.1186/s13068-016-0564-4 HMM_xylT_, HMM_gluT_ and HMM_MFS_ scores of the top 15 proteins, from the *A. niger* and *T. reesei* predicted proteomes.
10.1186/s13068-016-0564-4 Overview of transciptional regulation for the *A. niger* transporter proteins in the top scoring 25 % of HMM_xylT._

10.1186/s13068-016-0564-4 Growth curves of the Ag11C3 transformant strains expressing XltA, XltB, XltC, Str1, Str2 or Str3. The yeast transformants were grown on minimal medium with xylose (0.5 %; *w*/*v*) and xylose + glucose (0.5 + 0.5 %; *w*/*v*)_._

10.1186/s13068-016-0564-4 Graphical representations of the radiolabeled xylose and glucose initial uptake rates determined for the *A. niger* XltA, XltB and XltC; and the *T. reesei* Str1, Str2 and Str3 xylose transporters_._

10.1186/s13068-016-0564-4 Multiple sequence alignment (MSA) of all transporters used for HMM_gluT_ (constructed in a previous study, [9]) and HMM_xylT_ (this study), plus the newly identified *A. niger* and *T. reesei* transporters.
10.1186/s13068-016-0564-4 Primer sequences and plasmids used in this work.

## Abbreviations

HMM: hidden Markov model
MFS: major facilitator superfamily
SP: sugar porter
Hxt: hexose transporter
Gluc: glucose
Xyl: xylose
Soph: sophorose
NCS: no carbon source
LB: luria broth
protID: protein sequence identification number
OD: optical density
W: weight
V: volume
An: *Aspergillus niger*
Tr: *Trichoderma reesei*
ND: not detected
NC: not comparable
NQ: not quantifiable
FOA: 5-fluoroorotic acid
PBS: phosphate-buffered saline
MSA: multiple sequence analysis
RT-qPCR: reverse transcription-quantitative polymerase chain reaction
