# Supplementary material for: Identification and functional characterization of novel xylose transporters from the cell factories Aspergillus niger and Trichoderma reesei
Source: Biotechnol Biofuels. 2016 Jul 20;9:148. doi: 10.1186/s13068-016-0564-4 (PMC4955148; doi:10.1186/s13068-016-0564-4)
Supplement: Supplementary file 4 — 10.1186/s13068-016-0564-4 HMMxylT, HMMgluT and HMMMFS scores of the top 15 proteins, from the A. niger and T. reesei predicted proteomes. [file 13068_2016_564_MOESM4_ESM.pdf]

Results of the HMM searches

Top15 *A. niger* proteins - HMM_{xylT}

protID	HMM score	name
1143191	640.8	MstA
1143598	603.1	MstH
1169204	580.4	XltA
1142034	568.5	
1127588	563.6	XltB
1121621	558.9	
1180703	549.6	
1181257	539.2	
1142882	538.5	MstG
56566	535.5	
1167504	520.2	XltC
1125134	517.8	
1144375	514.9	
1101809	505.7	
1202517	504.4	

Top15 *A. niger* proteins - HMM_{xylT} excluding the proteins from *A. niger* and *T. reesei*

protID	HMM score	name
1143598	597.8	MstH
1169204	567.1	XltA
1143191	564.7	MstA
1142034	560	
1121621	557	
1127588	552.8	XltB
1180703	543.1	
1142882	539	MstG
1181257	534.4	
56566	529.6	
1167504	520.7	XltC
1125134	517	
1144375	514.9	
1101809	505.1	
1202517	496.6	

Top15 *A. niger* proteins - HMM_{MFS}

protID	HMM score	name
1187380	189.9	
1148471	176.8	
1123711	170.7	
1187333	170.3	
1217318	168.7	
1168101	167.6	
1107055	164.4	
1153334	160.7	
1101150	159.3	
1184384	158.3	
1200258	157.8	
1101864	157.6	
1123754	157.3	
1079444	156.3	
1183839	155.0	

Top15 *T. reesei* proteins - HMM_{xylT}

protID	HMM score	name
50894	661.7	Str1
63966	601.5	
121482	585.8	Str2
47710	571.4	Stp1
76800	570.5	
72383	562.6	
62380	536	Str3
106556	517.9	
22912	514.1	Hxt1
54005	510.4	
69026	509.5	
69901	500.8	
62502	492.3	
106330	476.7	
121850	476.3	

Top15 *T. reesei* proteins (HMM$_{xylT}$ excluding the proteins from *A. niger* and *T. reesei*)

protID	HMM score	name
50894	596.7	Str1
63966	596.3	
121482	584.1	Str2
76800	565.9	
72383	556.4	
62380	532.7	Str3
47710	525.2	Stp1
22912	515.2	Hxt1
106556	510.6	
54005	502.4	
69026	500.7	
69901	500.4	
62502	490.4	
121850	473.2	
106330	472.9	

Top15 *T. reesei* proteins - HMM$_{MFS}$

protID	HMM score	name
76641	191.9	
38341	180.3	
6103	173.9	
108893	170.2	
62488	160.9	
62171	158.8	
105798	156.2	
57749	155.2	
70829	154.5	
74953	153.0	
45523	152.7	
68925	152.6	
76775	152.0	
75937	148.1	
67692	148.1	
