# Supplementary material for: Identification and functional characterization of novel xylose transporters from the cell factories Aspergillus niger and Trichoderma reesei
Source: Biotechnol Biofuels. 2016 Jul 20;9:148. doi: 10.1186/s13068-016-0564-4 (PMC4955148; doi:10.1186/s13068-016-0564-4)
Supplement: Supplementary file 5 — 10.1186/s13068-016-0564-4 Overview of transciptional regulation for the A. niger transporter proteins in the top scoring 25 % of HMMxylT. [file 13068_2016_564_MOESM5_ESM.pdf]

Overview of transcriptional regulation for the *A. niger* transporter proteins in the top scoring 25% of HMM_{xylT}

Prot ID	Induced in xylose[a]	Induced in xylose + arabinose[b]	Induced in straw[c]	Induced in willow[d]	Induced in steam exploded sugarcane bagasse[e]	References
1169204-XltA	+	+	+	+	+	[30-33]
1142034					+	[30,32]
1167504 -XltC	+					[29]
1101809					+	[32]
1125086	+	+			+	[29,32]
1208766					+	[32]
1105490					+	[32]
1160647		+				[32]
1135963					+	[32]
1177862					+	[32]
1214934			+	+	+	[31,33]
1086238					+	[30]
1088440					+	[32]
1111761					+	[30,32]
1095309					+	[32]
1140337			+	+		[31,33]
1204192					+	[32]
1147409			+	+		[31,33]
1160976					+	[30]
1183460	+					[29]
1186625					+	[32]
1201932					+	[32]
1013169					+	[32]
1189278			+	+	+	[30,31,33]

a: significantly increased expression compared to the reference condition, containing sorbitol as single carbon source

b: significantly increased expression compared to the reference condition, containing fructose as single carbon source

c: significantly increased expression compared to the reference condition, containing glucose as single carbon source

d: significantly increased expression compared to the reference condition, containing glucose as single carbon source

e: significantly increased expression compared to the reference condition, containing fructose as single carbon source
