# Supplementary figures and images for: Identification and functional characterization of novel xylose transporters from the cell factories Aspergillus niger and Trichoderma reesei
Source: Biotechnol Biofuels. 2016 Jul 20;9:148. doi: 10.1186/s13068-016-0564-4 (PMC4955148; doi:10.1186/s13068-016-0564-4)

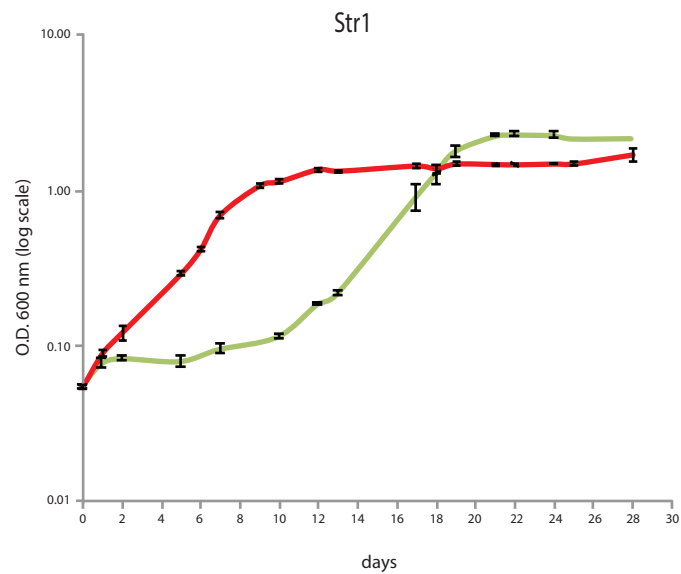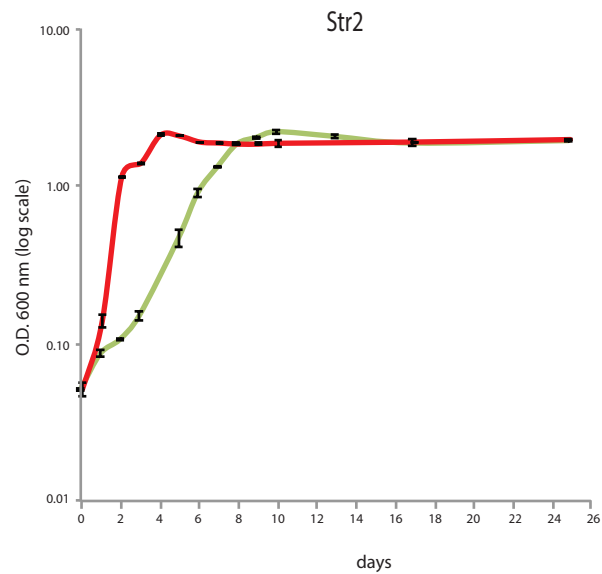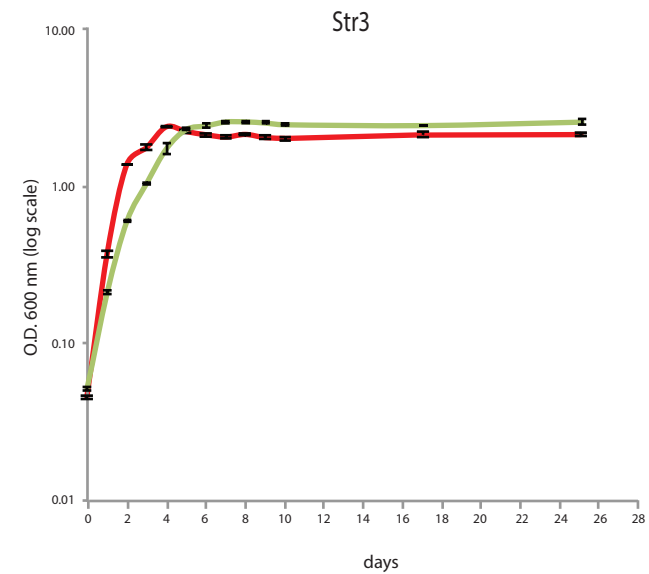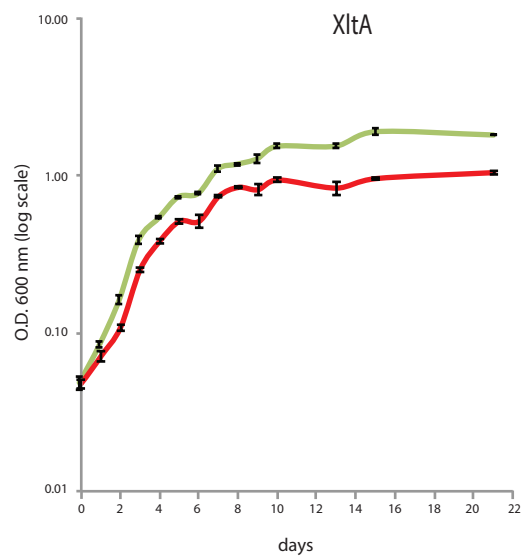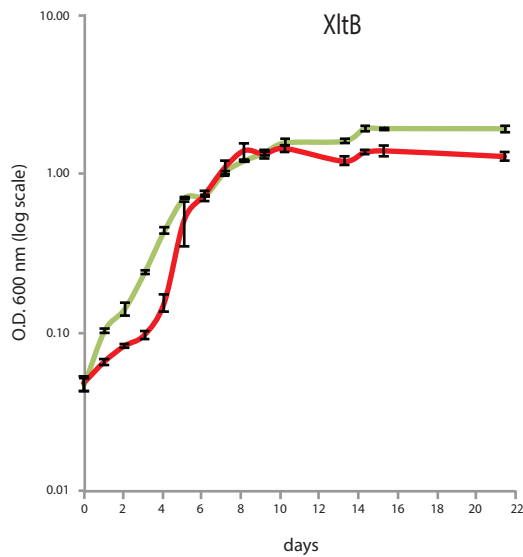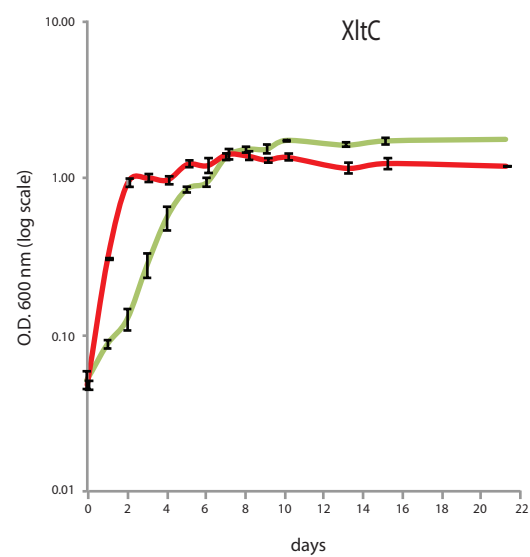

xylose 0.5%    xylose 0.5% + glucose 0.5%

Supplement: Supplementary file 6 — 10.1186/s13068-016-0564-4 Growth curves of the Ag11C3 transformant strains expressing XltA, XltB, XltC, Str1, Str2 or Str3. The yeast transformants were grown on minimal medium with xylose (0.5 %; w/v) and xylose + glucose (0.5 + 0.5 %; w/v). [file 13068_2016_564_MOESM6_ESM.pdf]

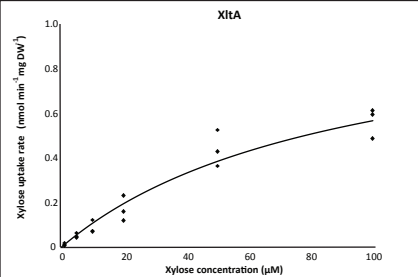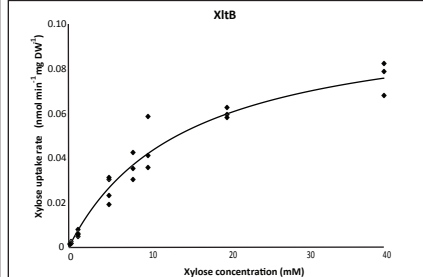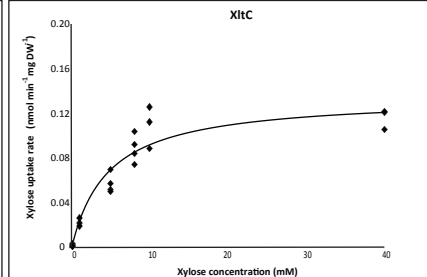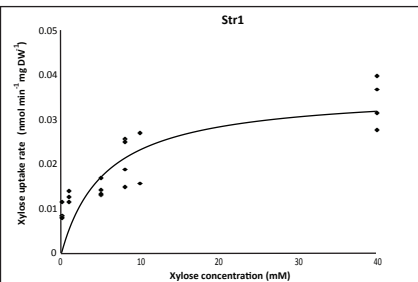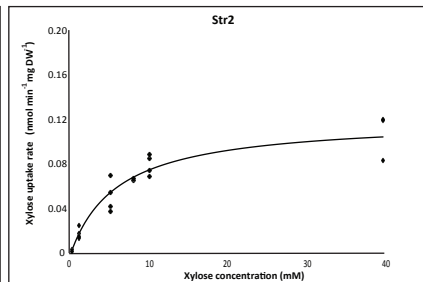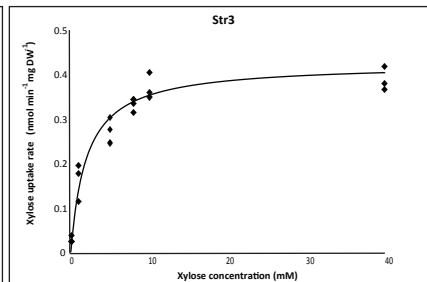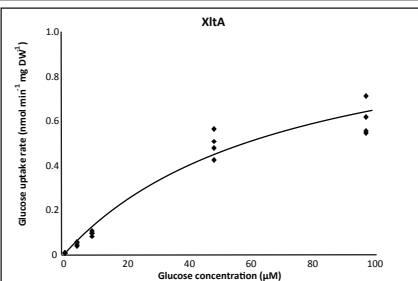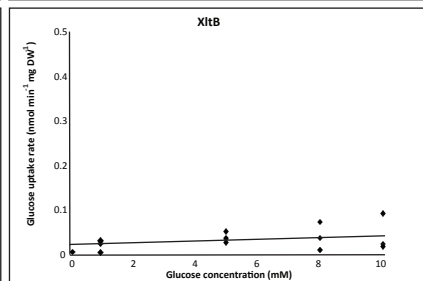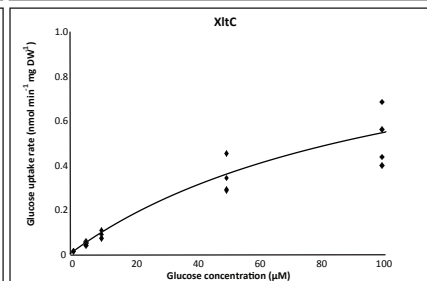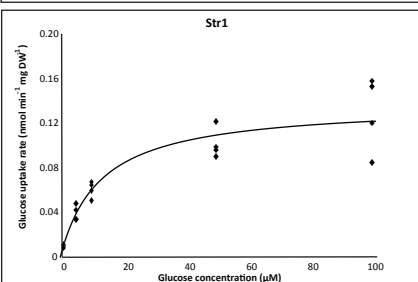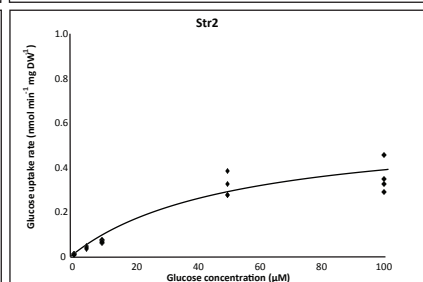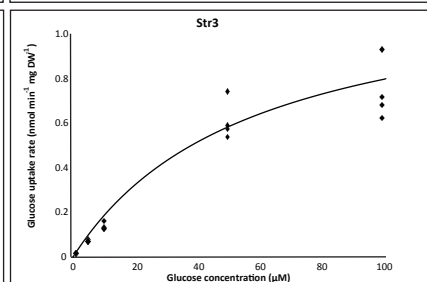

Supplement: Supplementary file 7 — 10.1186/s13068-016-0564-4 Graphical representations of the radiolabeled xylose and glucose initial uptake rates determined for the A. niger XltA, XltB and XltC; and the T. reesei Str1, Str2 and Str3 xylose transporters. [file 13068_2016_564_MOESM7_ESM.pdf]
