# Supplementary material for: Identification and functional characterization of novel xylose transporters from the cell factories Aspergillus niger and Trichoderma reesei
Source: Biotechnol Biofuels. 2016 Jul 20;9:148. doi: 10.1186/s13068-016-0564-4 (PMC4955148; doi:10.1186/s13068-016-0564-4)
Supplement: Supplementary file 9 — 10.1186/s13068-016-0564-4 Primer sequences and plasmids used in this work. [file 13068_2016_564_MOESM9_ESM.pdf]

Primers used for gene cloning into the plasmid p426HXT7-6His

Primer	Sequence (5'-3')
xltA_FW	GAGAACTAGTATGGGGATGGGTGCCGG
xltA_RV	GAGACTCGAGCTACGCCGAGGGAGGAGT
xltB _FW	CGATAAGCTTGATATCGAATTCCTGCAGCCCGGGGGATCCAGTCCTCGAGCCCTCACATGATGGATAAG
xltB _RV	ACTCACTATAGGGCGAATTGGGTACCGGGCCCCCCCTCGAGCGCATCGATTTAGGCCTCAATTACGG
xltC _FW	CGATAAGCTTGATATCGAATTCCTGCAGCCCGGGGGATCCATGCCTCGAGATGGCTATCGGCAATCTTTAC
xltC _RV	ACTCACTATAGGGCGAATTGGGTACCGGGCCCCCCCTCGAATCGATCACCTCAAGCAATCTTATCC
str1_FW	CAAAAAGTTAACATGCATCACCATCACCATCACACTAGTATGGGCGCGCACACCGAC
str1_RV	GACATAACTAATTACATGACTCGAGGTCGACGGTATCGATTCAAGCGGCCTTCTCAGC
str2_FW	CAAAAAGTTAACATGCATCACCATCACCATCACACTAGTATGTCGTCAAATCCAGCA
str2_RV	GACATAACTAATTACATGACTCGAGGTCGACGGTATCGATTCAGACATATTCTTCAGC
str3_FW	CAAAAAGTTAACATGCATCACCATCACCATCACACTAGTATGACCGTTACCTTCGAC
str3_RV	GACATAACTAATTACATGACTCGAGGTCGACGGTATCGATTTAGACATGCTCCTCGTG

The primer pair names indicate the amplified gene in each case

Primers used for RT-qPCR analysis

**Primer**	**Sequence (5'-3')**
xltA-RTqPCR_FW	TTCTACGACTACCTCTCGCTCGTC
xltA-RTqPCR _RV	AAAGCATGACCAAGCACGCAAC
xltB-RTqPCR _FW	AAGGTGGTGGACTCAAGAACGC
xltB-RTqPCR _RV	AGGGCATTGATTCCAACGAACTG
xltC-RTqPCR _FW	TGGTCGTCGTACTGCCATTCAG
xltC-RTqPCR _RV	ACAATGATGGATCCAATGCACCAG
hist-RTqPCR_FW	ATCTTGCGTGACAACATCCA
hist-RTqPCR_RV	CACCCTCAAGGAAGGTCTTG
str1-RTqPCR _FW	CACGTGCCACATCATCATCG
str1-RTqPCR _RV	CTCCCAGAGAAACACCATAAGG
str2-RTqPCR _FW	CGGAGTGTACGGAATTGTCAAC
str2- RTqPCR _RV	GGAGTAGGAGAAGTTGATGTCG
str3-RTqPCR _FW	ATCGTCTTCGCCTGTCTCTTC
str3-RTqPCR _RV	CAAAGTTGCAGGCAGCGAAG
sar1-RTqPCR_FW	TGGATCGTCAACTGGTTCTACGA
sar1- RTqPCR_RV	GCATGTGTAGCAACGTGGTCTTT
act1-RTqPCR_FW	TGAGAGCGGTGGTATCCACG
act1-RTqPCR_RV	GGTACCACCAGACATGACAATGTTG

The primer pair names indicate the amplified gene in each case

Plasmids used in this work

Name	Genotype	Description	Source/Reference
p426HXT7-6His	2μ, URA3, $HXT7_p$	Used as a vector for the expression of *A. niger* transporter genes, and as an empty vector (EV) for the construction of a *S. cerevisiae* negative control strain	[59]
pRH315	CEN6, ARSH4, TRP1	Plasmid expressing the *P. stipitis* D-xylose reductase (XYL1), the *P. stipitis* xylitol dehydrogenase (XYL2), and the *S. cerevisiae* xylulokinase (XKS1) genes. Used for the construction of a *S. cerevisiae* strain able to metabolize xylose.	[39]
p426HXT7-6His-xltA	2μ, URA3	Plasmid p426HXT7-6His expressing the *A. niger* transporter gene *xltA*	This study
p426HXT7-6His-xltB	2μ, URA3	Plasmid p426HXT7-6His expressing the *A. niger* transporter gene *xltB*	This study
p426HXT7-6His-xltC	2μ, URA3	Plasmid p426HXT7-6His expressing the *A. niger* transporter gene *xltC*	This study
p426HXT7-6His-str1	2μ, URA3	Plasmid p426HXT7-6His expressing the *T. reesei* transporter gene *str1*	This study
p426HXT7-6His-str2	2μ, URA3	Plasmid p426HXT7-6His expressing the *T. reesei* transporter gene *str2*	This study
p426HXT7-6His-str3	2μ, URA3	Plasmid p426HXT7-6His expressing the *T. reesei* transporter gene *str2*	This study
